# Long-Term Coexistence of Rotifer Cryptic Species

**DOI:** 10.1371/journal.pone.0021530

**Published:** 2011-06-28

**Authors:** Javier Montero-Pau, Eloisa Ramos-Rodríguez, Manuel Serra, Africa Gómez

**Affiliations:** 1 Institut Cavanilles de Biodiversitat i Biologia Evolutiva, Universitat de València, Valencia, Spain; 2 Department of Biological Sciences, University of Hull, Hull, United Kingdom; Biodiversity Insitute of Ontario - University of Guelph, Canada

## Abstract

Despite their high morphological similarity, cryptic species often coexist in aquatic habitats presenting a challenge in the framework of niche differentiation theory and coexistence mechanisms. Here we use a rotifer species complex inhabiting highly unpredictable and fluctuating salt lakes to gain insights into the mechanisms involved in stable coexistence in cryptic species. We combined molecular barcoding surveys of planktonic populations and paleogenetic analysis of diapausing eggs to reconstruct the current and historical coexistence dynamics of two highly morphologically similar rotifer species, *B. plicatilis* and *B. manjavacas*. In addition, we carried out laboratory experiments using clones isolated from eight lakes where both species coexist to explore their clonal growth responses to salinity, a challenging, highly variable and unpredictable condition in Mediterranean salt lakes. We show that both species have co-occurred in a stable way in one lake, with population fluctuations in which no species was permanently excluded. The seasonal occurrence patterns of the plankton in two lakes agree with laboratory experiments showing that both species differ in their optimal salinity. These results suggest that stable species coexistence is mediated by differential responses to salinity and its fluctuating regime. We discuss the role of fluctuating salinity and a persistent diapausing egg banks as a mechanism for species coexistence in accordance with the ‘storage effect’.

## Introduction

The last two decades have witnessed an increasing awareness of the widespread phenomenon of cryptic species [1]. These morphologically undistinguishable taxa have been described in almost all phyla, although they appear to be especially abundant among aquatic organisms [2]–[4]. The taxonomic uncertainty introduced by cryptic species has had a profound impact on various ecological fields: delaying the detection of biological invasions [5], [6], slowing down apparent evolutionary rates [7], and confounding the ecological niche of biological species [8]–[10]. Additionally, cryptic species pose a major challenge to other aspect of ecological theory. As they are so similar in their morphology and physiology a high degree of ecological similarity is expected [11]. Therefore, their existence in sympatry, which is common [e.g.], [ 10,12], poses a challenge regarding niche differentiation theory and the mechanisms that facilitate species coexistence.

Many studies have addressed the mechanisms responsible for coexistence in terrestrial [e.g.], [ 9,13] and marine sympatric cryptic species [e.g., 14]. In contrast, awareness of continental aquatic cryptic species is more recent and few studies deal with the ecological mechanisms mediating their coexistence [15]–[17]. The salt lake rotifer species *Brachionus plicatilis* and *B. manjavacas* share a virtually identical morphology and size. They belong to the *Brachionus plicatilis* species complex [3], [18] and, their species status has been supported by genetic and reproductive isolation analysis [18], [19]. Given their morphological similarity, the most reliable way to discriminate both species is molecular barcoding [20], [21]. *B. plicatilis* and *B. manjavacas* often co-occur in salt lakes in the Iberian Peninsula, where they are thought to have been present for at least several Pleistocene glaciations [22], [23]. Since *Brachionus* species reach very high population densities in short times in the field (for example, densities of thousands of individuals/L are commonly recorded for *B. plicatilis* in the Iberian Peninsula short after hatching [24], [25]), these species are likely to experience resource limitation in nature [26]. Laboratory experiments have shown competition between *B. plicatilis*, and *B. rotundiformis* and *B. ibericus*, which also belong to the same cryptic species complex but are smaller and morphologically different [27].

Six species of the *Brachionus plicatilis* cryptic species complex have been found living sympatrically in the Iberian Peninsula in inland salt lakes and coastal lagoons [23], [28], [29]. Until now, salinity and temperature [30], resource partitioning and differential vulnerability to predators [15], [27] have explained competitive outcomes for three of the species of the complex with the greater morphological and size differentiation (*B. plicatilis*, *B. ibericus* and *B. rotundiformis*). However, and given their high similarity in morphology and size, such factors appear unlikely to mediate *B. plicatilis* and *B. manjavacas* coexistence. Both *B. plicatilis* and *B. manjavacas* are osmoregulators [31] (and personal observation), and *B. plicatilis* population growth rate negatively affected by increasing salinity [31]. Since the lakes where these species co-occur in the Iberian Peninsula have a highly variable salinity regime [32], [33], it could be expected that salinity fluctuations affect the competition of both species.

Here, we explore the role of salinity fluctuations on the niche differentiation of *B. plicatilis* and *B. manjavacas*. We present data on (1) annual plankton population dynamics in two lakes, (2) historical population dynamics screening diapausing eggs from the sediment cores of a salt lake using a paleogenetic approach, and (3) laboratory growth rates at six salinities representative of the range experienced by both species in the wild. We provide evidence that both species co-occur in the water column and that salinity is a factor for niche differentiation. We propose that the fluctuating salinity regime can act as a stabilizing niche difference mediating their coexistence.

## Methods

### Annual population dynamics

The seasonal dynamics of *B. plicatilis* and *B. manjavacas* was studied in two Iberian salt lakes, Salobrejo and Pétrola (see Table 1), from October 2004 to April 2006. The lakes were visited every 2–3 weeks, and zooplankton samples taken whenever water was present. In each sampling event, salinity and conductivity were recorded using a WTW LF320 conductivity meter (Wissenschaftlich – Technische Werkstätten GmbH, Willheim, Germany). Quantitative samples were obtained to estimate rotifer density by collecting 6–30 L of lake water using a Van Dorn horizontal sampling bottle (6 L) and filtering them through a 30-µm Nytal mesh. When the lakes were too shallow to allow the sampling bottle to be used, samples were obtained by filtering lake water collected by repeatedly sweeping a 1 L plastic container until the sampling volume was reached. Zooplankton samples were fixed in situ with formaldehyde. Rotifer density in the quantitative samples was determined using a CK2 Olympus inverted microscope at 40×–100× magnification. All rotifers identified morphologically as belonging to the *B. plicatilis/B. manjavaca*s morphotype were counted.

**Table 1:** Lake information, maximum growth rates and optimal salinities.

			*B. plicatilis*		B. *manjavacas*	
Lake	Geographic location	Salinity range (g/L)	Salinity (g/L)	*r_max_*	Salinity (g/L)	*r_max_*-value
Hondo Sur	38°10′49″N, 0°45′19″O	8–18^1^^,^ 2	10	0.417	20	0.383
Manjavacas	39°25′00′N, 2°51′49″O	5–79^2^^,^ 3	10	0.313	10	0.336
Salobrejo	38°54′50′N, 1°28′11″O	8–65^1^^,^ 4	5	0.356	10	0.336
Pétrola	38°50′26′N, 1°33′56″O	10–280^4^^,^ 6	10	0.267	30	0.313
Balsa de Santed	41°00′58″N, 1°32′31″O	17–20^1^	10	0.300	-	-
Charca Universidad de Cádiz	36°32′02″N, 6°12′38″O	39^2^; 49^4^	5	0.466	-	-
Capacete	37°01′22″N, 4°49′36″O	3–6^5^	-	-	5–30*	0.267
Camino de Villafranca	39°21′45″N, 3°15′17″O	6–108^6^^,^ 7	-	-	10	0.363
Average optimal salinity			**7.5 g/L**		**17.5 g/L**	

*B. manjavacas grew with the same r_max_ from 5 to 30 g/L.

1 Lapesa [29];

2 Ortells et al. [28];

3 García-Ferrer et al. [62];

4 this study;

5 Rodríguez-Rodríguez & Moral Martos [63];

6 Boronat et al. [64];

7 Alonso [65].

Lakes where rotifer clones were isolated, with geographic location and their recorded salinity range. Salinity at which the maximum daily growth rate (*r_max_*) is achieved in each lake for *B. plicatilis* and *B. manjavacas*, as estimated in the laboratory, is shown. Average optimal salinity, calculated as the average of the salinity at which each clone reaches their maximum growth rate, is also provided.

Samples to estimate the relative abundances of *B. plicatilis* and *B. manjavacas* using molecular barcoding were collected using a 30 µm mesh plankton net, preserved in 70% ethanol and stored at 4°C in the dark until used. From each sample, up to 50 individuals with *B. plicatilis/B. manjavacas* morphology were randomly picked for species identification using PCR-RFLP of a mitochondrial gene fragment (cytochrome *c* oxidase, cox1, COI) following Campillo et al. (2005). In those samples with few individuals, all individuals were barcoded. This way, the relative abundance of each species in the lake was estimated.

### Long-term population dynamics

**Figure 1:**
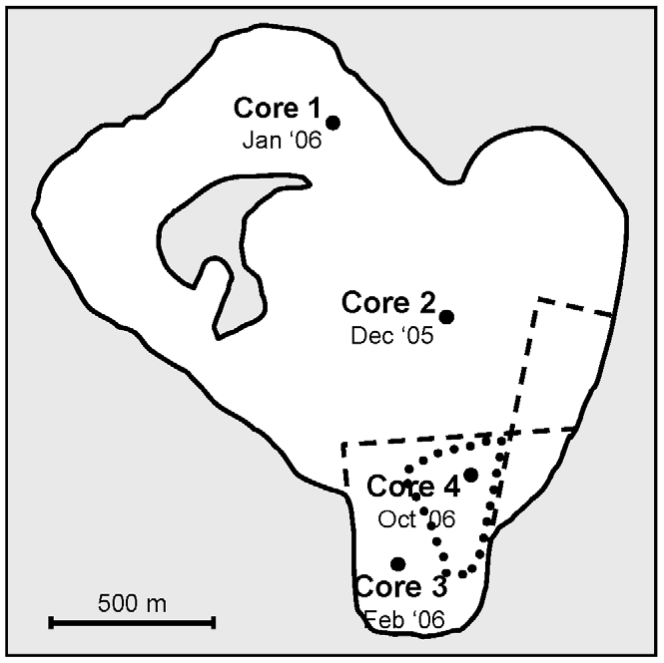
Location and date of sediment core sampling in Pétrola lake. Dashed lines show dikes of a disused salt evaporation plant that divide the lake into compartments. The more southern compartment receives the inflow of freshwater from a nearby sewage treatment plant. Dotted lines show the area with remaining water during a summer drought in August 2007 (from an aerial photograph in Google Earth 4.7). Note that Core 4 was obtained in the deeper point of the lake.

Four sediment cores of 57 mm diameter and 50 cm length were collected from Pétrola lake during December 2005–October 2006 (Fig. 1) using a piston core sampler (Eijkelkamp, Agrisearch Equipment). Cores were sliced into 0.5 cm width sections until a maximum depth of 10 cm, where diapausing egg bank had previously been shown to be extinct in this lake [34]. Each section was weighted and then stored in a Petri dish at 4°C in the dark until it was processed. Diapausing eggs were extracted from the sediment samples following García-Roger et al. [34], and, to establish whether cores were age-structured, each egg was assigned a degree of deterioration (i.e., on the proportion of egg occupied by the embryo) [35]. Eggs with 50% or more of volume filled by the embryo were considered suitable for genetic analyses and were isolated and kept at 60 g/L, 4°C in the dark to prevent hatching until DNA extractions were performed. Diapausing egg isolation was carried out in weighed subsamples of each sediment section until 30 eggs were isolated, until 2.5 g of sediment was processed without finding any eggs, or until 4 g of sediment was processed without finding any eggs suitable for genetic analyses. In cores 3 and 4, from depths 6.0-cm downwards we used a sucrose flotation technique [36] that allows to process larger volumes of samples, so all the sediment on each section could be processed. Due to the lower efficiency of this technique, extractions were repeated three times to ensure that all eggs were isolated. Diapausing egg densities were computed for each section.

For species identification in sediment cores (barcoding), we used PCR-Single Stranded Conformation Polymorphism (SSCP) analysis [37] of a 378 bp fragment of the mitochondrial gene 16S rRNA. SSCP allows processing a large number of samples in a more cost-effective way than PCR-RFLP. An average of 23 diapausing eggs was analyzed per section. DNA extractions were performed using a modified alkaline lysis protocol [38] in a final volume of 40 µL, and a fragment of the 16S rRNA gene was amplified using rotifer specific primers [39]. PCR reactions were performed in a final volume of 10 µL with 2 µL DNA, 1× (NH_4_)_2_SO_4_ buffer, 0.2 mmol/L of each deoxy nucleotide, 2.5 pmol of each primer and 0.15 U of *Taq* polymerase (Biotools), using the following PCR cycling conditions: 2 min at 94°C, 40 cycles of 30 s at 94°C, 30 s at 60°C and 40 s at 72°C and final extension of 3 min at 72°C. SSCP analysis was performed by mixing 2.5 µL of PCR product with 7.5 µL of denaturing buffer (95% formamide, 10 mmol/L sodium hydroxide, 0.25% bromophenol blue and 0.25% xylene cyanol) and incubating this mixture for 5 min at 95°C and transferring it immediately to a 4°C bath. Thirty-two denatured samples were loaded in a gel 0.5× MDE® (Cambrex Bio Science Rockland), 0.6× TBE (Merck), 10% glycerol, 0.05% TEMED and 0.1% ammonium persulfate) and run at 40 V and 4°C in 0.6× TBE (Merck) for 16 h. Gels were stained with 1× SYBR® Gold (Molecular Probes, Invitrogen). Each sample was assigned to an electromorph pattern by eye and one sample from each electromorph per gel was sequenced. An additional 10% of the samples on each gel were sequenced as quality control.

PCR amplifications for the samples selected for sequencing were repeated under the same conditions described above but in a final volume of 50 µl. Products were purified using High Pure PCR Product Purification Kit (Roche) and sequenced using ABI PRISM Dye Terminator Cycle Sequencing Ready Reaction Kit (Perkin-Elmer Biosystems) in both directions and run in an ABI 3700 sequencer (Perkin-Elmer Biosystems). Chromatograms were checked and edited using CodonCode Aligner v.1.6. (CodonCode Corporation).

We used a generalized linear model [40] with a binomial distribution and a logit link function to test the effect of sampling point (core) and depth on the relative frequency of *B. plicatilis* and *B. manjavacas*. Tests were performed with R v.2.7.1 (R Development Core Team 2006).

### Effect of salinity on growth rate

Clonal growth rates of six *B. plicatilis* and six *B. manjavacas* clones were estimated in the laboratory at six salinities (5, 10, 20, 30, 40 and 45 g/L). Clones were obtained by hatching diapausing eggs isolated from sediment samples of eight Iberian salt lakes covering a wide range of salinity conditions (see Table 1). Experimental salinities were chosen according to this range and data in the literature, suggesting dramatic growth rate decrease, even negative growth, in the range 40–50 g/L for different clones of these species [41]. Clones of both species could be obtained for four lakes (Manjavacas, Pétrola, Salobrejo and Hondo Sur) (Table 1). Clones were identified at species level by PCR-RFLP of a COI fragment [20]. All clones except one from Charca Universidad de Cádiz and another from Capacete had been obtained and identified as part of another study [42].

Clonal growth rate was estimated in 216 cultures (2 species×6 clones×6 salinities×3 replicates). Pre-experimental cultures were started by placing approximately 650 females of each rotifer clone in 2 L diluted artificial seawater (Instant Ocean®, Aquarium Systems) at 25 g/L salinity (i.e., the intermediate experimental salinity), and 25°C in the dark. Rotifers were fed inert cells of the microalgae *Tetraselmis suecica* (see below). The experimental cultures were set up by placing 12 egg-bearing females from each pre-experimental culture into a 6-cm diameter Petri dish with 50 ml diluted seawater at the experimental salinity and adding 100 µL of a suspension of 10^5^ cells/mL inert cells of *T. suecica*. Cultures were kept in the dark at 25°C. Female rotifers in each experimental culture were counted at days 2 and 4, and exponential clonal growth rates (*r*) were estimated as *r* = (ln *N_4_*−ln *N_2_*)/2, where *N_2_* and *N_4_* are the number of female rotifers in the second and fourth day, respectively.

To produce the inert microalgal cells, two 5 L cultures of *T. suecica* were grown in diluted seawater fertilized with f/2 medium [43], at 25 g/L salinity, 25°C and 35 µmol quanta m^−2^ s^−1^ (constant illumination). After 10–12 days, the cultures were concentrated by centrifugation (12.6 g for 5 min), and thoroughly mixed. 20 mL aliquots of 10×10^6^ cells/mL for the pre-experimental cultures and 1.2 mL aliquots of 50×10^6^ cells/mL for the experimental cultures were prepared and kept at −80°C until needed. This procedure allows us to control for food quality variation since a single algae stock is used throughout the experiment.

A three-way, fixed effect ANOVA was performed to test for the effect of salinity on clonal growth rates for each species. Kolmogorov-Smirnov test for normality and Brown-Forsythe [44] test of homogeneity of variances were used to check the fit of data to ANOVA assumptions. Additionally, the response of each species to salinity was explored by fitting a quadratic function, where the independent variable was the *r* values averaged over replicates and clones, and the independent variable was salinity. Fitting was computed using least-squares regression. A quadratic function was selected because it is a simple one able to detect an intermediate maximum for the independent variable, accordingly with the expectation for the response to an environmental condition. All statistical tests were carried out using SSPS v 12.0.1 (SPSS Inc., Chicago, IL).

## Results

### Annual population dynamics

**Figure 2:**
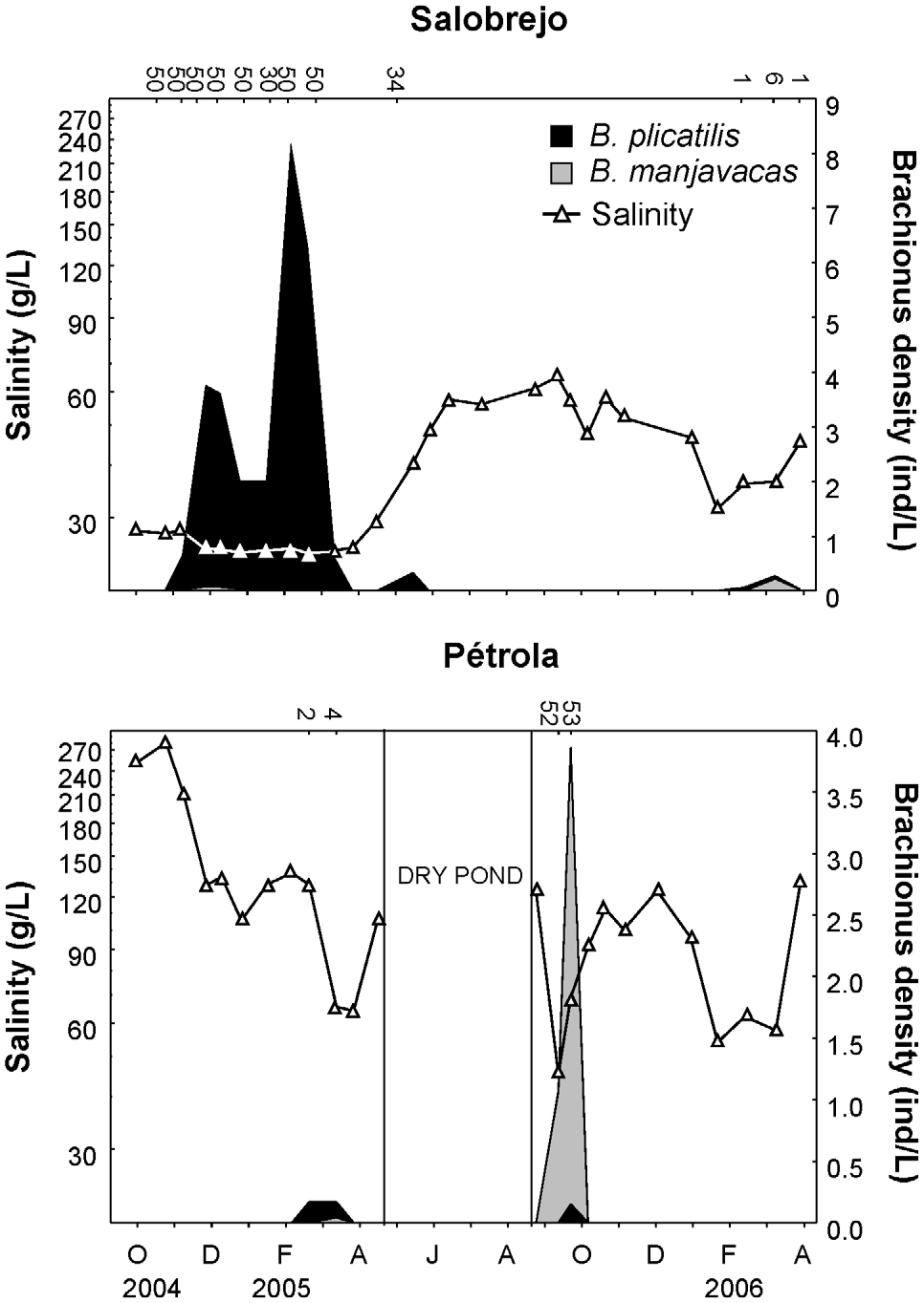
Salinity variation and population density. Salinity variation and population densities of *B. plicatilis* and *B. manjavacas* from October 2004 to April 2006 in Salobrejo and Pétrola lakes. Density estimates are based on the relative frequencies of the two species analyzed by RFLP. Numbers on top are the number of individuals analyzed. Note that population density scale is different in each graph.

During the study period, Pétrola and Salobrejo lakes showed wide salinity fluctuation and both reached hypersaline conditions. Salinity variation was more pronounced in Pétrola, which also reached much higher values than Salobrejo (Fig. 2). *B. plicatilis* and *B. manjavacas* showed very low prevalence and abundance in both lakes (Fig. 2) and populations appeared to grow opportunistically, with population peaks occurring during narrow time windows of lower salinity. *B. plicatilis* was detected in 14 and *B. manjavacas* in 10 out of 55 samples. Both species were found together in eight samples, although one of them was always much more abundant than the other. A contrasting pattern was found in the abundance of both species between the two lakes; in Salobrejo, *B. plicatilis* was the most abundant species, while *B. manjavacas* was the abundant species in Pétrola. In Salobrejo, *B. plicatilis* was detected when salinity decreased to around 25 g/L (from November 2004 to March 2005). In Pétrola, *B. manjavacas* was present in the water column only when salinity decreased to 45 g/L during a very short period of time (from September to October 2005). These results suggest that *B. plicatilis*, if compared to *B. manjavacas*, has a temporal distribution associated to low-range salinity periods in these lakes.

Other rotifer species (*Brachionus quadridentatus*, *Lecane* sp., *Notholca* sp. and *Keratella* sp.), a species of cladoceran and two species of copepods were also present in the water column in both lakes. Maximum densities of these species were lower than those of *B. plicatilis* and *B. manjavacas*; they ranged from 0.08 to 2.63 individuals/L in Pétrola lake and from 0.03 to 5.18 individuals/L in Salobrejo lake. The only exception was one of the copepod species in Salobrejo lake that reached a maximum density of 198 individuals/L.

### Long term population dynamics

A total of 9244 diapausing eggs belonging to the *B. plicatilis* species complex were isolated from the four sediment cores and classified according to their degree of deterioration. Total diapausing egg bank density (i.e., eggs in any deterioration state and empty egg shells) varied from 31.97 to 73.12 eggs/cm^3^ for the upper first cm and from 7.93 to 23.47 eggs/cm^3^ for the upper ten cm. Viable eggs - i.e., those eggs with more than 75% of the space occupied by the embryo, which make up most of the diapausing eggs that contribute to the hatchings [35] - are 46.0–79.8% of the eggs in the upper 1-cm layer. Cores 1 and 2 were explored until a maximum depth of 7 cm where the egg bank was considered to be extinct, while densities in Cores 3 and 4 remained of about 5 eggs/cm^3^ in the deepest section analyzed (10.0–10.5 cm).

Both total diapausing egg and viable egg densities showed a clear negative relationship with depth in all four cores, although Cores 3 and 4 showed a subsurface peak of density at around 2.25 cm and 7.25 cm respectively. In consequence, the diapausing egg bank showed a pattern of increased deterioration with depth. Both results suggest an age-structured egg bank. Although no data are available for Pétrola, sedimentation rates in other similar inland salt lakes of Eastern Iberian are about 0.05–0.35 cm/year [34]. Using this range of sedimentation rates, we estimated an approximate maximum age of 28.5 to 200 years for the first 10 cm. In addition, as the four cores were taken in different sites in the lake basin (see Fig. 1) they are likely to be affected by different hydrological and salinity regimes and thus, their sedimentation rates may differ. For example, Cores 3 and 4 were taken in the south side of the lake, separated from the north by a dike of a disused salt evaporation plant, and with a freshwater inflow from a nearby village sewage treatment plant; consequently, its salinity and hydrological regime differ from the north side.

**Figure 3:**
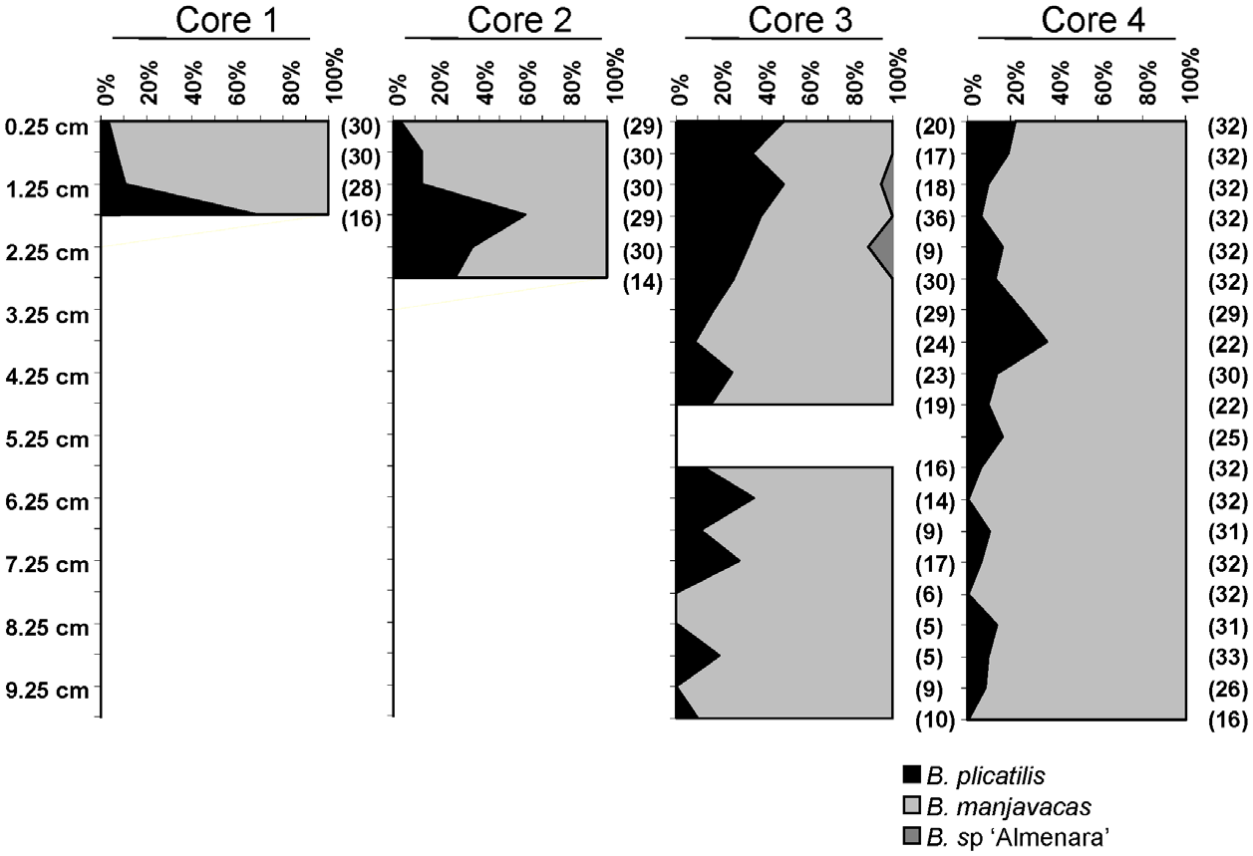
Species relative abundance along the four sediment cores. Relative abundance along the four cores of *B. plicatilis*, *B. manjavacas* and *B.* sp ‘Almenara’. Numbers in brackets are sample sizes of diapausing eggs. Section 5.25 cm of Core 3 could not be analyzed due to failed DNA extractions. Dashed lines mark 50% of abundance.

A total of 1167 diapausing eggs were considered suitable for genetic analysis and were analyzed by SSCP. Of these, 255 were sequenced (GeneBank accession numbers JN035646–JN035900). Six different SSCP electromorphs corresponding to six 16S rRNA haplotypes were found, three of them belonging to *B. plicatilis*, two to *B. manjavacas* and one to *B. sp* ‘Almenara’ (a species of the *B. plicatilis* complex not formally described yet). All haplotypes found in the subsample of randomly sequenced diapausing eggs corresponded with those predicted by the SSCP analysis. There was a clear numerical dominance of *B. manjavacas* over *B. plicatilis* in the four sediment cores (Fig. 3), although this situation was reversed in some sediment depths. Despite *B. plicatilis* being rarer, it was detected consistently, being only absent in 5 samples out of 49, three of them with low sample size. There is no observable trend towards the exclusion of any of the species at any given depth. The generalized linear model comparing the relative frequency of both species showed significant differences among cores (p<0.001), depths (p<0.001), and an interaction effect depth×core (p<0.001). Deviations accounting for the interaction effect show that *B. plicatilis* is more frequent than expected in the southern side of the lake (Cores 3 and 4), with lower salinity, while the opposite is found in the northern side (Cores 1 and 2). Diapausing eggs of *B.* sp. ‘Almenara’ appeared twice in Core 3 at depths 1.25 and 2.25 cm, reaching an abundance of 5.6% and 11.1% respectively, and coinciding with an increase in the abundance in *B. plicatilis* in the same core.

Differences in the variance of the logarithm of the recruitment are informative on competition dynamics [45] (see below for further details). Therefore, we computed the variance of the ln of the total diapausing egg density for each core, assuming an exponential loss of eggs in the sediment for each slice, and we found that *B. plicatilis* showed a higher variance of the estimated recruitment than *B. manjavacas*, with an average difference for the four cores of 1.47±0.45. Results for the different cores are: Core 1, 1.68 vs. 0.30 (F_3,3_ = 0.178, p-value = 0.190); Core 2, 1.03 vs. 0.17 (F_5,5_ = 0.112, p-value = 0.032); Core 3, 1.66 vs. 0.06 (F_18,18_ = 0.034, p-value<0.001) and Core 4, 1.99 vs. 0.01 (F_19,19_ = 0.006, p-value<0.001). Note that when density for any species was zero, it was replaced by the minimum density recorded in all the cores (0.064 eggs/cm^3^), as a conservative approach. Similar qualitative results were obtained when no correction for egg loss in the sediment was used or different kinds of eggs were considered (i.e., with/without empty eggs; data not shown).

### Effect of salinity on growth rate

**Figure 4:**
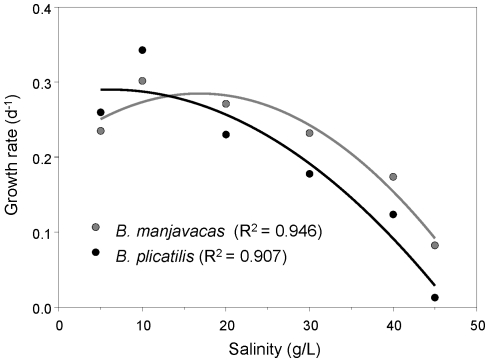
Relationship between mean *r* values and salinity. Relation between mean *r* values, after averaging over clones and replicates, of *B. plicatilis* and *B. manjavacas*, and salinity. Curves are least-squares quadratic functions. Determination coefficients (R^2^) are shown.

The analysis of variance revealed substantial variation in growth rates in response to salinity (Table 2). *B. manjavacas* tended to have higher growth rates than *B. plicatilis*, growth rates averaged over clones and salinities being 0.216 and 0.191 days^−1^ respectively. This might reflect the effect of a pre-adaptation to the conditions in our experiments (e.g., temperature, food conditions, etc.). All 12 clones displayed positive mean growth rates at all but the highest salinity tested (45 g/L). The highly significant interaction between species and salinity (Table 2) indicates that both species differed in their response to salinity. Depending on the clone, *B. plicatilis* had their highest *r* at 5 or 10 g/L, while the ones for *B. manjavacas* were in the range 10–30 g/L. Within-species variation in optimal salinity, as assessed by the maximum *r*, was wider in *B. manjavacas* than in *B. plicatilis* (Table 1). Values of *r* averaged over clones and replicates, and then fitted to quadratic functions point out the overall trend in the response of these species to salinity (Fig. 4). *B. manjavacas* had higher mean *r* than *B. plicatilis* at 20–45 g/L salinity, while the opposite was observed at 5–10 g/L. Moreover, at 45 g/L, three *B. plicatilis* clones (from Manjavacas, Pétrola and Universidad de Cádiz lakes) showed negative growth rates, but only one of *B. manjavacas* (from Camino de Villafranca lake) did it.

**Table 2:** ANOVA results of the effects of lake, species and salinity on the clonal growth rate (*r*).

Source of variation	df	*F*	p-level
Lake	7	9.121	<0.001
Species	1	38.933	<0.001
Salinity	5	142.448	<0.001
Lake×Species	3	1.926	0.128
Lake×Salinity	35	11.540	<0.001
Species×Salinity	5	4.440	0.001
Lake×Species×Salinity	15	4.293	<0.001
Error	144		

## Discussion

We have shown that *B. plicatilis* and *B. manjavacas* have co-occurred in a stable way in a lake, with long-term population fluctuations in which no species was permanently excluded. Both species have differential growth rates in response to salinity, with *B*, *plicatilis* growing better at lower salinities than *B. manjavacas*. In addition, our field study reports for the first time quantitative evidence for the co-occurrence of *B. plicatilis* and *B. manjavacas* in the water column. Since *B. plicatilis* and *B. manjavacas* have co-occurred in the Iberian Peninsula from the Pleistocene, altogether these data suggest that the coexistence of these two morphologically identical species in the area is persistent, and not due to a lasting, but transient random walk towards the extinction of one of the species.

Populations of both species were found in the periods of lower salinity in both lakes, being *B. manjavacas* more abundant in Pétrola, the lake with a higher salinity, while the opposite pattern was found in Salobrejo, which had lower salinity. The sporadic occurrence and low population densities achieved were probably due to the extremely high salinity reached as a consequence of the drought during the sampling period. Rainfall during 2005 in the area is amongst the lowest since 1940 (data from Spanish Agencia Estatal de Meteorología). In fact, *B. plicatilis* and *B. manjavacas* were the most abundant species in the zooplankton during the studied period, only exceeded by a species of copepod in Salobrejo. In wetter years, individuals with *B. plicatilis/B. manjavacas* morphotype have been found in Salobrejo and Pétrola at much higher population densities (187 and 68 individuals/L) [46], which makes competition a feasible scenario. Nevertheless, our data illustrates that even a short time window of low salinity may offer environmental conditions for opportunistic growth of these species. We do not find evidence for seasonal succession, although we cannot rule it out.

Our results suggest that subtle differential responses to salinity play a role in niche differentiation of these two species. In other species of the *B. plicatilis* species complex, salinity is associated to their differential spatial and temporal distribution [17], [47]. In particular, *B. manjavacas* tends to occur in hypersaline lakes [22]. In agreement with this, our results show that there is a consistent trend for *B. manjavacas* to occur at higher salinities than *B. plicatilis* in the field. It supports our laboratory results showing an average higher salinity optimum for *B. manjavacas* than for *B. plicatilis* clones. As salinity tolerance ranges overlap, this factor will not be directly limiting species occurrence, but affecting their relative fitness, which has implications for competition. The role of salinity as a challenging factor for these species is also supported by physiological data. *B. plicatilis*, and most likely *B. manjavacas*, is an osmoregulator [31 and personal observation]; i.e. the higher the salinity, the more resources need to be allocated to maintain the internal osmotic pressure.

In absence of historical limnological studies, paleolimnological analysis can provide a proxy for the long-term coexistence dynamics of species in a lake. Our combination of paleolimnology and paleogenetic analysis of Pétrola lake sediment cores, which have signatures of being age-structured, supports the absence of a replacement trend between *B. plicatilis* and *B. manjavacas* for at least several decades and possibly centuries. Correlations between species densities in the sediment and past densities in the water column are however not straightforward, as they depend on taxon-specific recruitment to and from the egg bank and differences on egg preservation [48], [49]. Although no published data exist on egg production or preservation for these species, our long experience culturing both species does not indicate differences in diapausing egg production per female. In addition, differences in deterioration rates in sediment cores of diapausing eggs of species of the *B. plicatilis* complex in different lakes were mainly due to lake sediment conditions regardless the species composition [34]. All this, together with the highly similar morphology of *B. plicatilis* and *B. manjavacas* diapausing eggs, supports that the relative frequencies of their diapausing eggs in the sedimentary record are likely to give information on past species fluctuations in recruitment. Accordingly, *B. manjavacas* appears as the most abundant species in this lake, although *B. plicatilis* was able to reverse this situation several times along the recent history of the lake. The increases of *B. plicatilis* diapausing egg recruitment could have been associated with reductions of the lake salinity during periods of higher rainfall, which is supported by the association of an increase of *B. plicatilis* in Core 3 with the presence of *B.* sp ‘Almenara’, a species associated with low salinities [17]. Core 3 was taken in a shallow part of the southern, lower salinity area of the lake, and given the correlation of salinity with depth in salt lakes [33] this core is likely to reflect recruitment during periods of lower salinity. In a similar way, the differential response to salinity of both species is also supported by the much higher frequency of *B. manjavacas* in both cores from the more saline north side of the lake.

We conclude that *B. plicatilis* and *B. manjavacas* have coexisted and coexist in a stable regime in the Iberian Peninsula. If, as expected, resource competition between them occurs, one or several stable coexistence mechanisms should be regulating their population dynamics. Mechanisms such as resource partitioning through food particle size or food quality, or differential predation vulnerability appear unlikely, due to the extreme resemblance in size and shape of the external morphology and the grazing and trophic structures [21] of these two suspension-feeding rotifers. In addition, the salt lakes where these species coexist in the Iberian Peninsula are shallow and offer few opportunities for microhabitat differentiation. However, salinity fluctuates highly and unpredictably at a large range of temporal scales in salt lakes (from seasonal to interannual) as a consequence of meteorological and climatic changes [33]. This is specially marked in the Iberian Peninsula, due to its Mediterranean climate, with remarkably large and largely unpredictable inter-annual fluctuations in rainfall [32], [33], [50], and to the effect of global climatic phenomena such as El Niño-Southern Oscillation events, which have particularly strong effects in the SE, where the studied lakes are located [51]. Environmental fluctuations are a key part of several stable coexistence mechanisms [52], [53]. One of such mechanisms is the ‘storage effect’, based on recruitment fluctuation, which has been suggested to be a coexistence mediating mechanism in many systems [45]. The storage effect is also likely to mediate species coexistence in continental zooplankton -e.g., copepods, cladocerans and rotifers- [e.g.], [ 54,55]. However, until now, just one study has demonstrated the storage effect in these organisms [56]. Our results allow a preliminary exploration of this mechanism for coexistence of *B. plicatilis* and *B. manjavacas*

Three components are needed to create the necessary population feedback that allows a competing species to recover from low densities in a fluctuating environment [52]: (1) a life-cycle stage buffered from competition; (2) a differential response of the competing species to a fluctuating environment; and (3) covariance between environment and competition. Of these three components, the first and the second are met by *B. plicatilis* and *B. manjavacas*. Both species produce diapausing eggs – which do not compete for resources –, and we have shown here that they have a differential response to salinity, a highly fluctuating condition in the lakes where they coexist; with *B. manjavacas* outperforming *B. plicatilis* in the higher salinity range. We cannot rule out that instead of salinity, other physical environmental factors correlated to it might also contribute to this differential response. The third component measures the population response to the physical environment and acts as a stabilizing mechanism; the environment, by altering differentially the population density, modifies the competition, i.e. the species favored by the environmental conditions suffers from a greater competition. The greater the magnitude of the environment-competition covariance, the lower the benefits are for the species when the environment is favorable and the greater the opportunity for a competitor species. Showing that this component actually happens has been proposed as a rigorous field test of the storage effect [57]. However, to date, no experimental data exists assessing this component in temporal fluctuating environments. An indirect approach based on the comparison of the variance of ln recruitment (‘recruitment variation’) of the species has been developed to test this component and, thus, the storage effect [45]. The test is based on the fact that a high environment-competition covariance results in a low variation in recruitment. Therefore, a difference in the recruitment variation between competitors is expected if a difference in environment-competition covariances occurs, as required by the storage effect. As explained in Chesson [45], the difference in recruitment variance between competing species is proportional to the community storage effect; and this comparison reflects the full magnitude of the storage effect in an invader-resident scenario, where the invader species will have a higher variance. That scenario can be expected when comparing a situation of high versus low-density species [45]. This is a sound assumption for our system (Fig. 3), with *B. manjavacas*, the most abundant species in the paleolimnological record, being the resident species, and *B. plicatilis* the invader. Additionally, Chesson [45] also recognizes that this scenario is expected when studying small fast growing organisms, which is the case of our species. Our data for the four cores show that *B. plicatilis* always has a higher estimated recruitment variance than *B. manjavacas*, suggesting an environment-competition covariance. Therefore, our data are what we should expect if the storage effect is a factor mediating the coexistence of *B. plicatilis* and *B. manjavacas*.

Notwithstanding the storage effect associated to salinity fluctuation, other coexistence mechanisms could be interacting with it in stabilizing coexistence. Little explored ecological factors (e.g., oxygen concentration, pH or ionic composition) could also be involved in niche differentiation. In addition, mechanisms not based in niche differentiation such as density-dependent life-cycle switching [58] are likely.

Despite the increasing use of paleolimnology in ecological and evolutionary studies [e.g.], [ 5], [59,60], this approach had not been used, to our knowledge, to document long-term coexistence of cryptic species before. Our results conclusively show long-term coexistence in of *Brachionus plicatilis* and *B. manjavacas*, two highly similar rotifer species, although with fluctuations in their relative densities. We have also shown that these species have different but overlapping responses to salinity. We propose that stable species coexistence is mediated by such differential responses to salinity in the context of habitat fluctuations. As King [61] stated almost three decades ago, “the ‘population’ investigated in many [limnological] studies may be an artifact with closer affinities to griffins, unicorns and mermaids than to the population as a biological unit”. Our work contributes to the increasing awareness that molecular techniques, by unveiling hidden species richness, can reveal concealed past and present ecological and evolutionary patterns.
